# Biomechanical investigation of positive reduction in the femoral neck fracture: a finite element analysis

**DOI:** 10.3389/fbioe.2024.1374299

**Published:** 2024-10-09

**Authors:** Xiang Zhou, Xishan Li, Kai Oliver Böker, Arndt F. Schilling, Wolfgang Lehmann

**Affiliations:** ^1^ Department of Trauma Surgery, Orthopedics and Plastic Surgery, University Medical Center Göttingen, Göttingen, Germany; ^2^ Department of Articular and Traumatic Orthopedic Surgery, Fourth People’s Hospital of Guiyang, Guiyang, Guizhou, China

**Keywords:** femoral neck fracture, finite element analysis, positive reduction, quantitative analysis, biomechanical investigation

## Abstract

**Background:**

Gotfried positive reduction offers an alternative strategy for femoral neck fracture (FNF) when achieving anatomical reduction is challenging. However, the biomechanical consequences of positive reduction remain unclear. The purpose of this study was to investigate the biomechanical behavior of positive reduction across different Pauwels classification, providing a reference for quantifying positive reduction in clinical practice.

**Methods:**

Three-dimensional (3D) models of FNF were established and categorized according to the Pauwels classifications (Pauwels I, II, and III), each of them contained seven models with different reduction qualities, including an anatomical reduction model, two negative reduction models, and four positive reduction models, all of which were stabilized with dynamic hip screws (DHS) and cannulated screws (CS). We investigated the maximal von-Mises stress of internal fixation and proximal femoral, femoral fragment displacement, and maximal von-Mises strain at the proximal fragment fracture site when a 2100 N load was applied to the femoral head.

**Results:**

The maximum von-Mises stress on the internal fixators in each Pauwels group was lowest in the anatomical reduction model. In the Pauwels I group, positive reduction exceeding 3 mm resulted in the maximum von-Mises stress on the internal fixators surpassing that of the negative reduction model. For the Pauwels II group, positive reduction beyond 2 mm led to the maximum von-Mises stress on the internal fixators exceeding that of the negative reduction model. In the Pauwels III group, positive reduction beyond 1 mm caused the maximum von-Mises stress on the internal fixators to be higher than that of the negative reduction model. The maximum von-Mises strain at the fracture site of proximal femur fragment increased with positive reduction. Varus displacement increased in positive reduction models as the Pauwels angle rose, potentially exacerbating rotation deformity in Pauwels III group.

**Conclusion:**

Excessive positive reduction may increase the risk of FNF failure after internal fixation. From a biomechanical stability perspective, positive reduction should be limited to 3 mm or below in the Pauwels I group, restricted to not exceed 2 mm in the Pauwels II group, and should not exceed 1 mm in the Pauwels III group. Negative reduction should be avoided in all Pauwels groups.

## 1 Introduction

**FIGURE 1:**
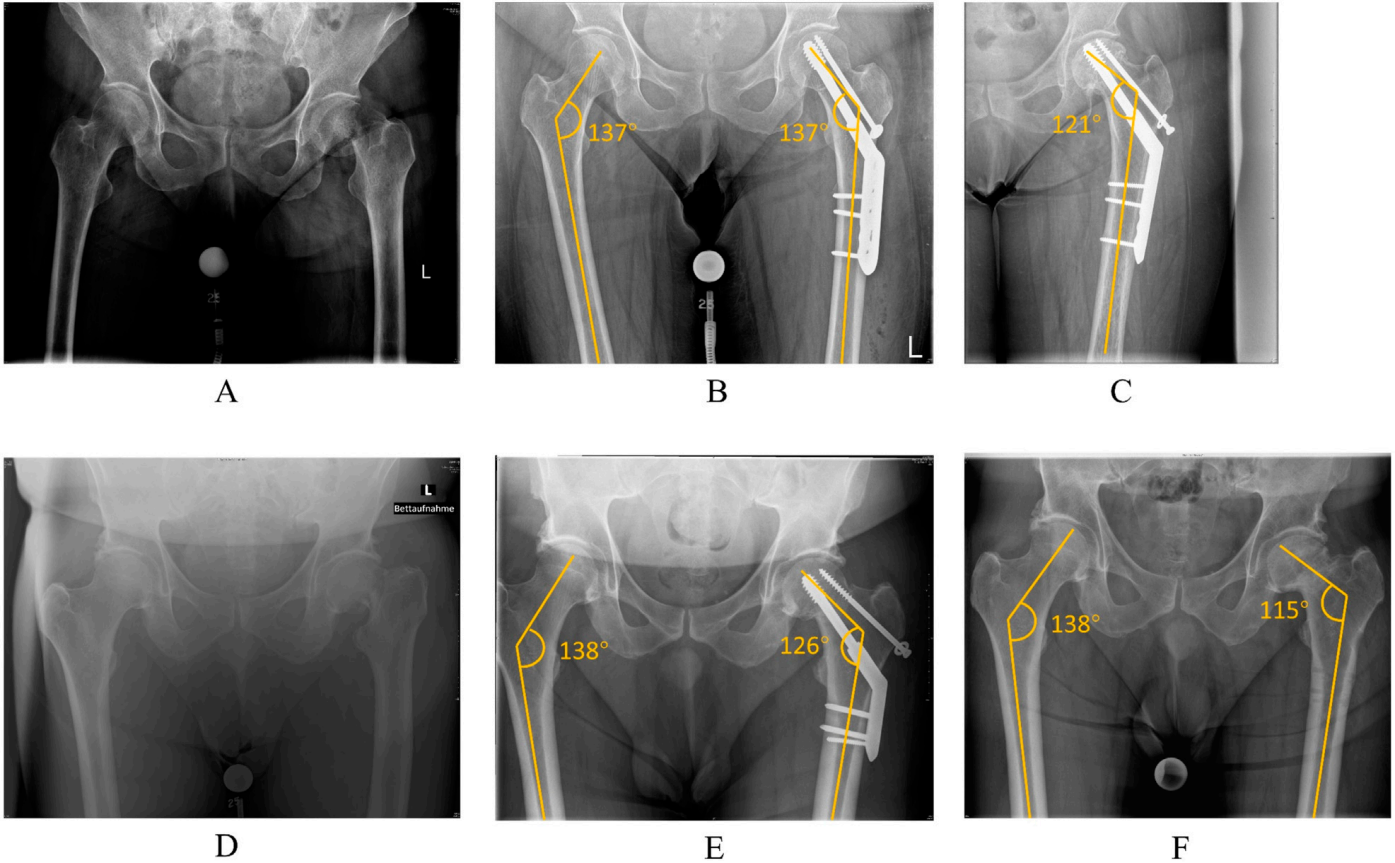
**(A)** The Pauwels III classification of left femoral neck fracture. **(B)** Treated with positive reduction and DHS + CS internal fixation, restoring the left femoral neck shaft angle to 137°, the same as the healthy side. **(C)** The left femoral neck shaft angle reduced to 121° with an obvious hip varus deformity at 1-year follow-up. **(D)** The Pauwels III classification of left femoral neck fracture. **(E)** The patient was treated with positive reduction and DHS + CS internal fixation. **(F)** The left femoral neck shaft angle reduced to 115° with an obvious hip varus deformity when the fracture was united, and the internal fixation was removed.

The femoral neck fracture (FNF) is a common joint trauma, accounting for about 3.58% of all body fractures and about 50% of proximal femur fractures (Wang Y. et al., 2019). High energy trauma is the most common reason for FNF in young patients. However, an excellent reduction, firm internal fixation, and as much preservation of the hip joint as possible have been the primary goals of treatment (Slobogean et al., 2015; Kang et al., 2016). Previous studies have shown the importance of anatomical reduction for FNF prognosis (Ly and Swiontkowski, 2009; Stacey et al., 2016; Slobogean et al., 2017). Nevertheless, repetitive repositioning to achieve perfect anatomical reduction may destroy the residual blood supply of the femoral head and lead to femoral head necrosis (Ghayoumi et al., 2015). In 2013, Gotfried introduced the concept of positive reduction for FNF, suggesting that it could achieve clinical outcomes similar to anatomical reduction (Gotfried et al., 2013). In his study, he elaborated on the following concepts: Positive reduction refers to the AP view of the distal femoral neck fragment positioned to the inferior medial margin of the proximal femoral neck fracture fragment. Negative reduction refers to the AP view of the distal femoral neck fragment positioned to the superior lateral margin of the proximal femoral neck fracture fragment. Therefore, positive reduction is acceptable when anatomical reduction is challenging to perform. In contrast, negative reduction should be avoided as much as possible because it often predicts a higher rate of postoperative complications (Zhao et al., 2021a; Zhao et al., 2021b). Thus, after the theory of positive femoral neck reduction has been proposed, many scholars have studied the mechanics of this theory and observed the clinical outcomes. A retrospective study reported that a combination of internal fixation with Gotfried positive reduction could be an effective treatment in young patients with some advantages such as improved mechanical support, reduced surgical time, decreased radiation exposure, and higher rates of excellent-to-good outcomes based on the Harris hip score (Zhu et al., 2022). Furthermore, some scholars concluded that the positive reduction demonstrated a comparable incidence of reoperations as anatomic reduction of FNF, and the positive reduction may lead to similar clinical results with anatomical reduction, but the negative reduction should be avoided (Xiong et al., 2019; Huang et al., 2020; Zhao et al., 2021a). However, from these studies and our clinical cases, we can notice that there are instances of complications such as hip varus and reoperations, even in some cases that underwent positive reduction (Figure 1). To further explore the mechanical properties of positive reduction, some studies have suggested that the extent of positive reduction should be confined to below 3 mm in Pauwels I FNF (Wang G. et al., 2019). Moreover, Fan et al. assumed that positive reduction is more stable than negative reduction when the Pauwels angle was at 30°, however, this advantage weakened when the Pauwels angle reached 50° (Fan et al., 2023). Although these studies have discussed the characteristics of positive reduction of FNF, there is still a lack of a mechanism analysis on the biomechanical behaviour with different reduction configurations based on the Gotfried positive reduction concept, especially for the Pauwels II and III groups. This study aims to explore the biomechanical behavior of positive reduction in various Pauwels classifications, providing a reference for quantifying positive reductions in clinical practice and helping to avoid the overuse of positive reduction in the treatment of FNF.

## 2 Materials and methods

### 2.1 Establishing three dimensional FNF models

**FIGURE 2:**
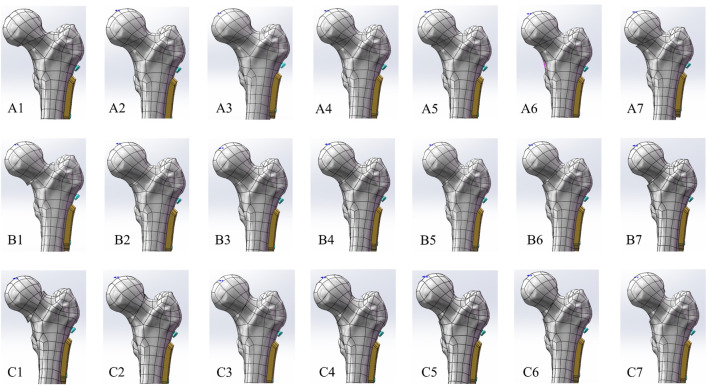
Based on the theory of Pauwels classification established the different FNF reduction models. **(A)** Pauwels I group (30°): A1) negative reduction 2 mm model, A2) negative reduction 1 mm model, A3) anatomical reduction model, A4) positive reduction 1 mm model, A5) positive reduction 2 mm model, A6) positive reduction 3 mm model; A7) positive reduction 4 mm model; **(B)** Pauwels II group (50°): B1) negative reduction 2 mm model, B2) negative reduction 1 mm model, B3) anatomical reduction model, B4) positive reduction 1 mm model, B5) positive reduction 2 mm model, B6) positive reduction 3 mm model; B7) positive reduction 4 mm model; **(C)** Pauwels III group (70°): C1) negative reduction 2 mm model, C2) negative reduction 1 mm model, C3) anatomical reduction model, C4) positive reduction 1 mm model, C5) positive reduction 2 mm model, C6) positive reduction 3 mm model; C7) positive reduction 4 mm model.

The anonymized computed tomography (CT) data utilized in this study originated from a 50-year-old male patient and was obtained from the Department of Radiology at the University Medical Center Göttingen. The CT data, free of deformities or pathologies, underwent processing through 3D Slicer software (version 5.0.2, https://www.slicer.org) and Geomagic Wrap software (3D Systems Corporation, United States) to create a detailed 3D model of the left femur, with cortical bone thickness specified as 5 mm (Li et al., 2020). The FNF models of Pauwels I (30°), Pauwels II (50°), and Pauwels III (70°) were reconstructed in SolidWorks 2018 (Dassault Systèmes Corporation, United States) based on the theory of Pauwels definition (Pauwels, 1958). According to the previous study, anatomical reduction was the golden standard for treating femoral neck fractures (Slobogean et al., 2017). Furthermore, due to both the higher possibility of complications and failure rates, the negative reduction for treating femoral neck fracture was not accepted in clinical practice (Zhao et al., 2021a). Therefore, we utilized the anatomical reduction model in each group as the compared model, and based on the positive reduction theory, we rebuilt the different distances of the positive reduction model (1, 2, 3, 4 mm) to investigate and quantify the positive reduction. Meanwhile, to illustrate the biomechanical significance of the medial inferior cortical buttress and estimate the biomechanical behavior of negative reduction for treating the femoral neck fracture, we built the negative reduction model (1, 2 mm) in each group as the negative compared group (Figure 2). Specifications for cannulated screws (CS) and dynamic hip screws (DHS) were determined based on prior studies (Wang et al., 2021), the specifications for the CS are as follows: a thread diameter of 7.3 mm and a hollow diameter of 2.9 mm and referred to the different reduction model to select the optimal length. For the DHS, the parameters are as follows: a plate thickness of 5.8 mm, a width measuring 17.4 mm, dynamic compression hip screws exhibiting a diameter of 12.7 mm, a thread length spanning 22 mm, and based on the various reduction situation to modify the optimal length. The 3D models of FNF fixed with DHS combined with CS were constructed following the Association for the Study of Internal Fixation (AO/ASIF) procedure. The constructed 3D model was then imported into ANSYS 2021R2 software (ANSYS Corporation, United States), which was used to obtain the finite element model.

### 2.2 Material properties

The femoral and the internal fixation were assumed to be homogeneous and isotropic with linear elastic properties that were reported by the previous studies, and the material of DHS and CS was assumed to be the titanium alloy (Cui et al., 2022; Fan et al., 2023). The Young’s modulus and Poisson’s ratios of these materials that were utilized in this study are shown in Table 1.

**TABLE 1:** The Young’s modulus and the Poisson’s ratios of materials in our study.

Material	Young’s modulus (MPa)	Poisson’s ratio
Cortical bone	16,800	0.29
Cancellous bone	840	0.29
Ti-6Al-7Nb	110,000	0.33

### 2.3 Contact conditions

In this FE analysis, we assumed that the contact conditions were different between the different components of the FNF model. The bonded contact was used to simulate the interaction between the cortical and cancellous, the thread of CS and DHS with cortical, the locking screws and the plate of DHS, and the locking screws with both cortical and cancellous. The frictional contact was used to these interfaces between the lateral cortical of the proximal femoral shaft and the DHS and CS with a friction coefficient of 0.3, and the contact interfaces between the dynamical compression screw of DHS, CS, and cancellous bone with a friction coefficient of 0.3 (Panteli et al., 2015). The frictional contact interface between the fragments has a friction coefficient of 0.46 (Yang et al., 2013).

### 2.4 Boundary and loading conditions

**FIGURE 3:**
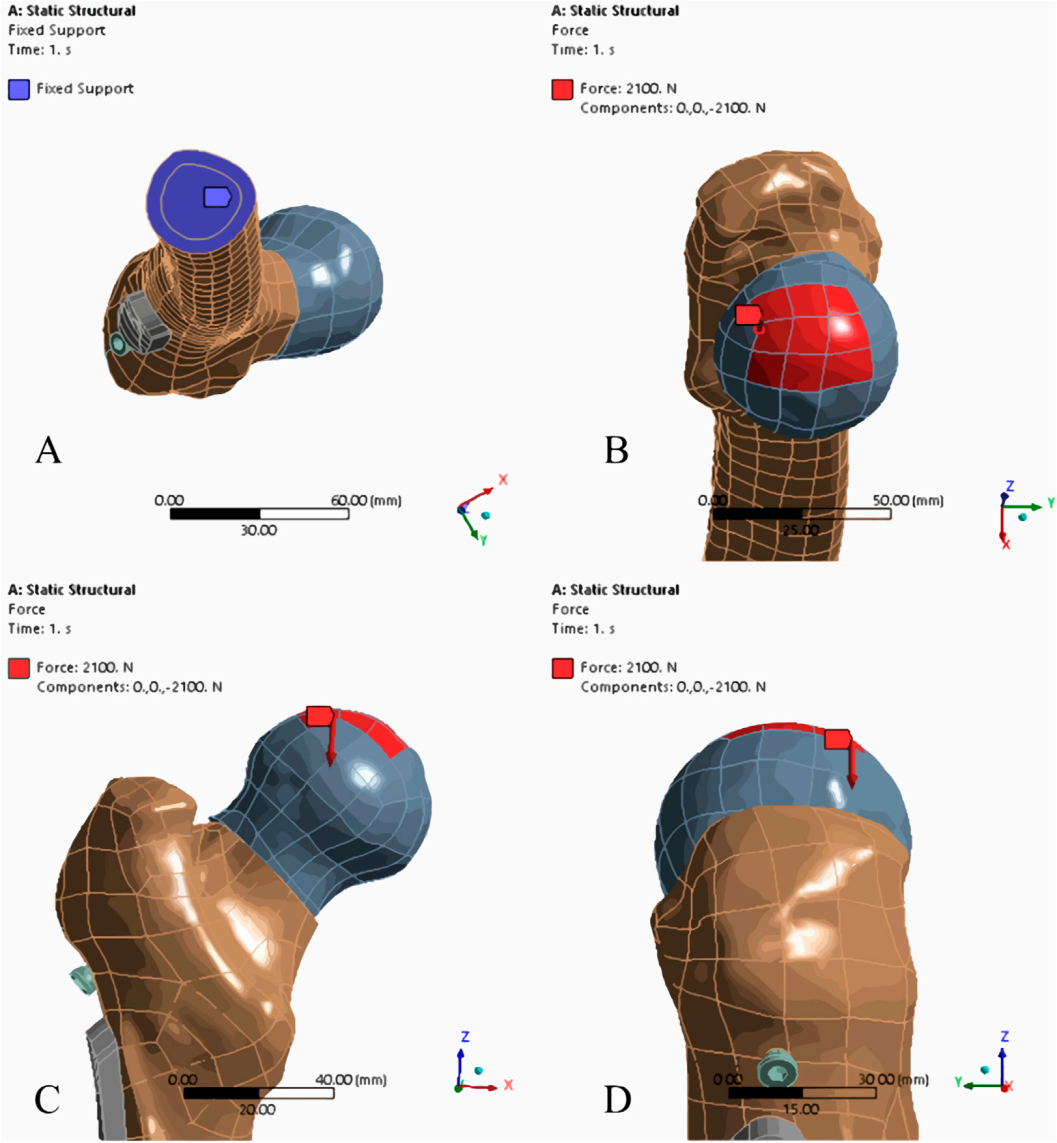
Boundary and loading conditions. **(A)** The blue region represents the area where the distal femur was immobilized in all directions by the constraint; **(B)** The red region at the top of the femoral head represents the area subjected to applied force; **(C, D)** Illustrate the force application direction in both coronal **(C)** and sagittal **(D)** planes, where in the direction of force application is congruent with the mechanical axis of the lower limb, consists with the negative direction of the *Z*-axis in the default coordinate system.

**FIGURE 4:**
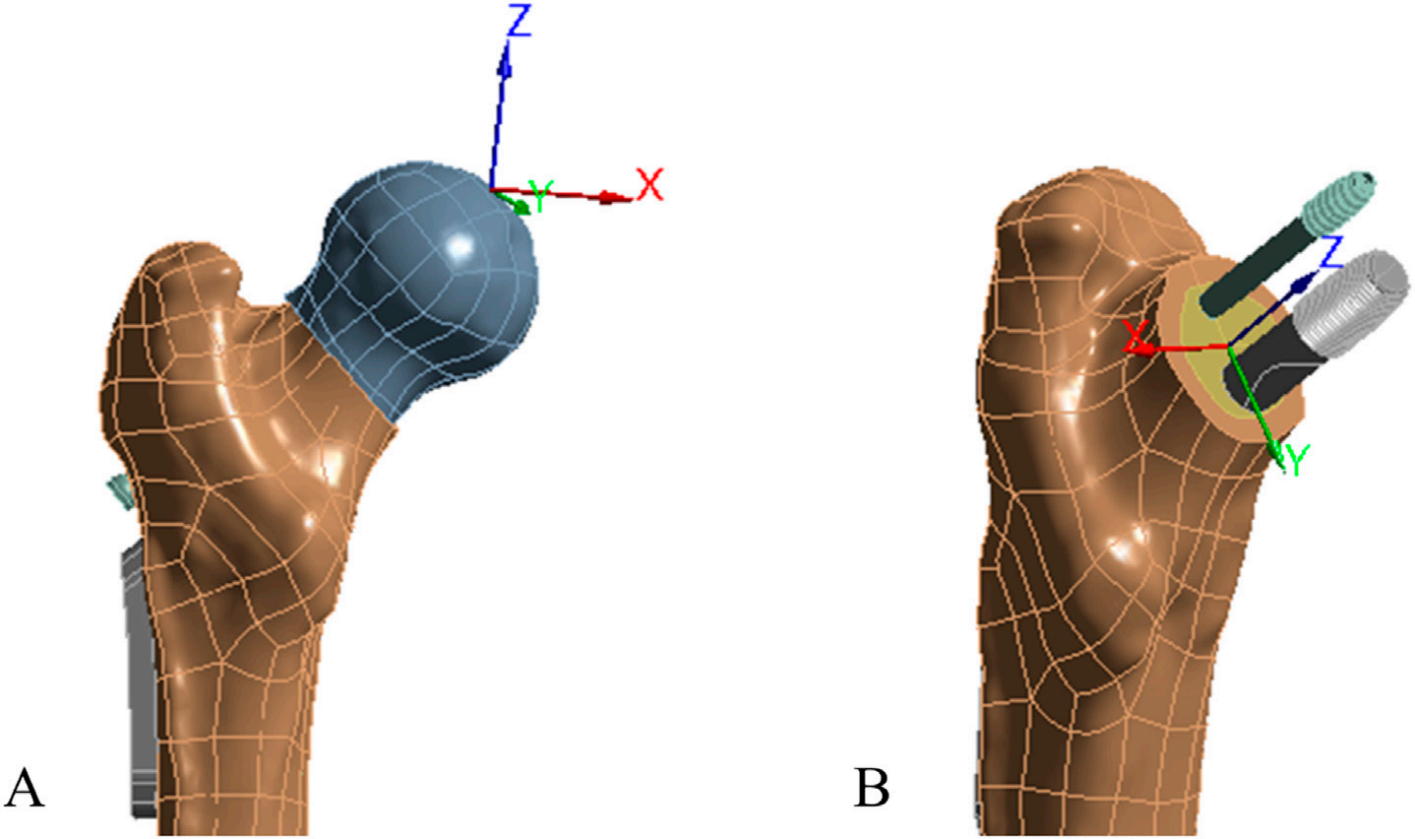
Established the regional coordinate system of proximal fragment. **(A)** femur mechanical axis coordinate system; **(B)** designating the center of the distal fracture surface as the coordinate origin; utilizing the line that parallel to the fracture surface and points to the center of medial inferior margin of the distal fracture site as the *Y*-axis; establishing the line that vertical to the *Y*-axis and points to the center of femoral head as the *Z*-axis; the *X*-axis is defined as the line that vertical to both *Y* and *Z*-axis and points to the posterior of femoral.

To imitate the typical human physiological standing position, the inferior surface of the femur within the experimental model is steadily fixed. Concurrently, a force of 2100 N that is equivalented three times a 70 kg person’s body weight and aligned with the mechanical line of the femur is applied on the top of the femoral head, as depicted in Figure 3. Due to the proximal fragment of the femoral being the most unstable part under load conditions, in order to investigate the displacement trend of the proximal fragment under the load, we established a regional coordinate system for assisting the displacement of the proximal femoral fragment in the three-dimensional directions. As illustrated in Figure 4, the *X*-axis direction was perpendicular to both the *Y* and *Z*-axes, pointing towards the posterior aspect of the femur, indicating the potential rotational trend of the femoral head fragment. The *Y*-axis direction was parallel to the fracture surface, pointing towards the center of the medial inferior margin of the distal fracture site, representing the potential trend of vertical displacement of the femoral head fragment. The *Z*-axis direction was perpendicular to the *Y*-axis, pointing towards the center of the femoral head, indicating the potential trend of varus of the femoral head fragment.

### 2.5 Meshing

To ensure the accuracy and stability of the FE analysis results, we conducted a mesh independence test. In this study, the initial mesh division used tetrahedral elements with an average grid size of 3.0 mm for the DHS and CS, and 4.5 mm for the femur. Based on this, the mesh was further refined using grid sizes of 2.5 mm, 2.0 mm, and 1.5 mm for the DHS and CS, and 4.0 mm, 3.5 mm, and 3.0 mm for the femur. To ensure the convergence of results, maximum von Mises stress and maximum displacement were selected as key physical quantities to compare their changes at different mesh densities. Table 2 presents the calculated results of maximum von Mises stress and maximum displacement at different mesh densities. As the mesh was progressively refined, the results gradually stabilized. When the mesh size was refined from 2.0 mm to 1.5 mm for the DHS and CS, and from 3.5 mm to 3.0 mm for the femur, the change rate of the maximum von Mises stress was less than 0.5%, and the change rate of the maximum displacement was less than 0.2%, indicating that the solution had converged. The mesh independence test indicates that at this mesh density, the finite element model’s computational results possess sufficient accuracy and stability. The resultant experimental model encompasses a comprehensive three-dimensional finite element model constituted of 363,610 nodes and 227,829 elements.

**TABLE 2:** The result of mesh independence test.

	Mesh size (mm)	Maximum von-mises stress (MPa)	Rate of stress change (%)	Maximum displacement (mm)	Rate of displacement change (%)
CS and DHS	3.0	158.37	-	5.9011	—
	2.5	160.16	1.13	5.873	0.47
	2.0	161.7	0.96	5.9085	0.60
	1.5	161.11	0.36	5.8974	0.18
Femur	4.5	158.37	-	5.9011	—
	4	160.16	1.13	5.873	0.47
	3.5	161.7	0.96	5.9085	0.60
	3.0	161.11	0.36	5.8974	0.18

### 2.6 Evaluation criteria

Our investigation included five parameters: the von-Mises stress of internal fixators and the proximal femur, the von-Mises strain at the fracture site of the proximal fragment, displacement of the femur and displacement of the femoral head fragment under regional coordinate system. When evaluating the stability of FNF reduction models post-internal fixation, the efficacy of the internal fixation device predominantly influences the stability of the fractured site before fracture union. In this study, we considered the maximum von-Mises stress on the internal fixation as the primary indicators for assessing stability with different reduction models.

### 2.7 Model validation

Most FE analysis studies validate their models using the same indicators to compare with numerous previous studies. Due to the scarcity of FEA studies using the same indicators for the intact proximal femur, our study assessed the intact femur FE model based on its maximum von Mises stress, axial stiffness, and von Mises stress at eight points on the femoral neck section. These three indicators were compared to the results of previous studies and served as the foundation for validating the credibility of the FE model developed in this study (Papini et al., 2007; Zhang et al., 2009; Fu et al., 2012; San Antonio et al., 2012; Miura et al., 2017; Chen et al., 2019; Jian-Qiao Peng et al., 2020; Wang et al., 2021).

## 3 Result

### 3.1 The maximum von-mises stress on the internal fixators

**FIGURE 5:**
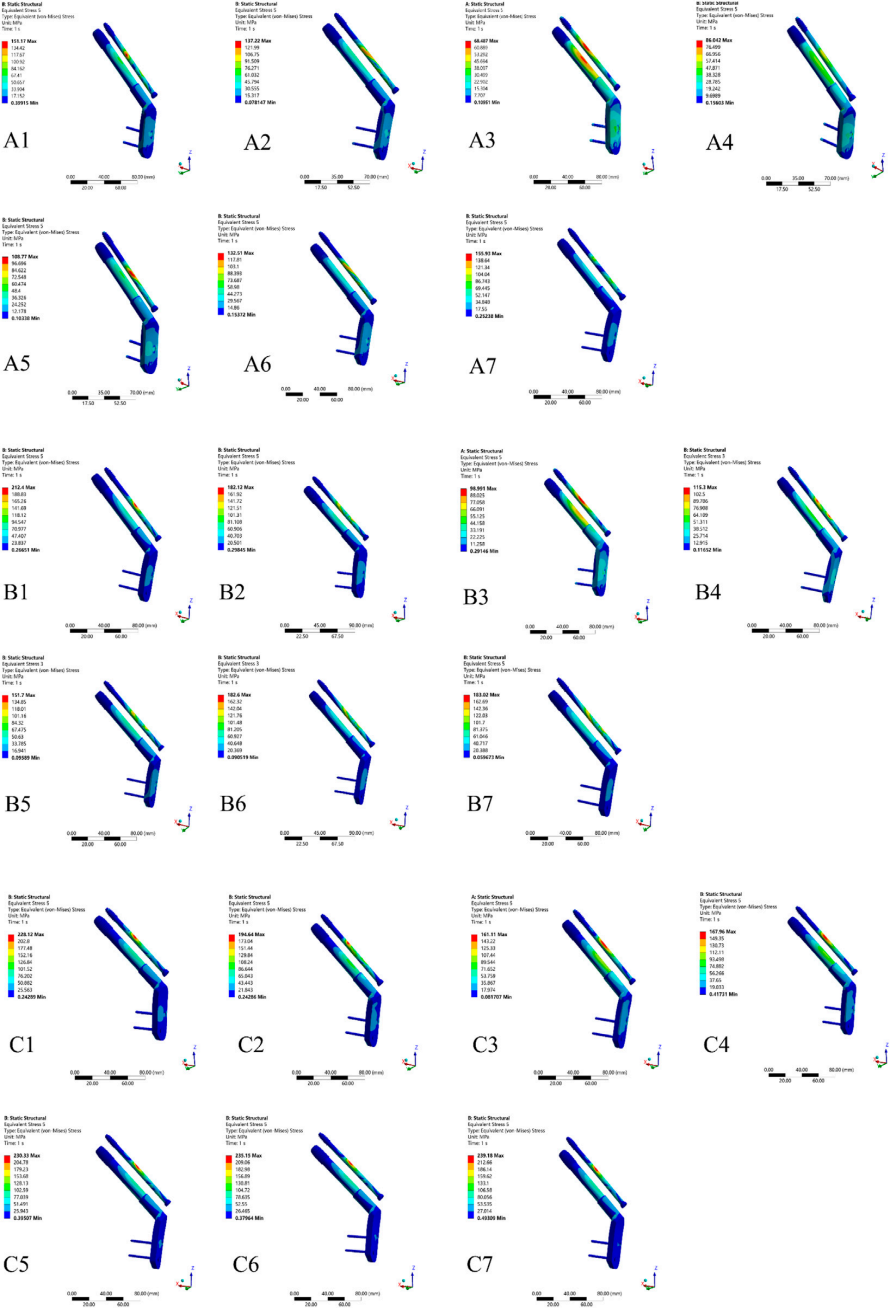
Maximum von-Mises stress nephograms of DHS and CS in different Pauwels group with different reduction models. **(A)** Maximum von-Mises stress nephograms of Pauwels I group: A1-A2) negative reduction 2 mm and 1 mm model, A3) anatomical reduction model, A4-A7) positive reduction 1 mm, 2 mm, 3 mm and 4 mm model; **(B)** Maximum von-Mises stress nephograms of Pauwels II group: B1-B2) negative reduction 2 mm and 1 mm model, B3) anatomical reduction model, B4-B7) positive reduction 1 mm, 2 mm, 3 mm and 4 mm model; **(C)** Maximum von-Mises stress nephograms of Pauwels III group: C1) negative reduction 2 mm and 1 mm model, C3) anatomical reduction model, C4-C7) positive reduction 1 mm, 2 mm, 3 mm and 4 mm model.

As the von-Mises stress nephograms shown in Figure 5, in the Pauwels I and II models, the internal fixator stress of different reduction qualities of FNF appeared to be concentrated on the CS and the compression screw of DHS located on the fracture line of the femoral neck and evenly distributed on the screw. However, in the Pauwels III models, the internal fixator stress associated with varying reduction qualities of FNF was primarily focused on the CS and secondary focus on the compression screw of DHS, which were situated on the fracture line of the femoral neck.

**FIGURE 6:**
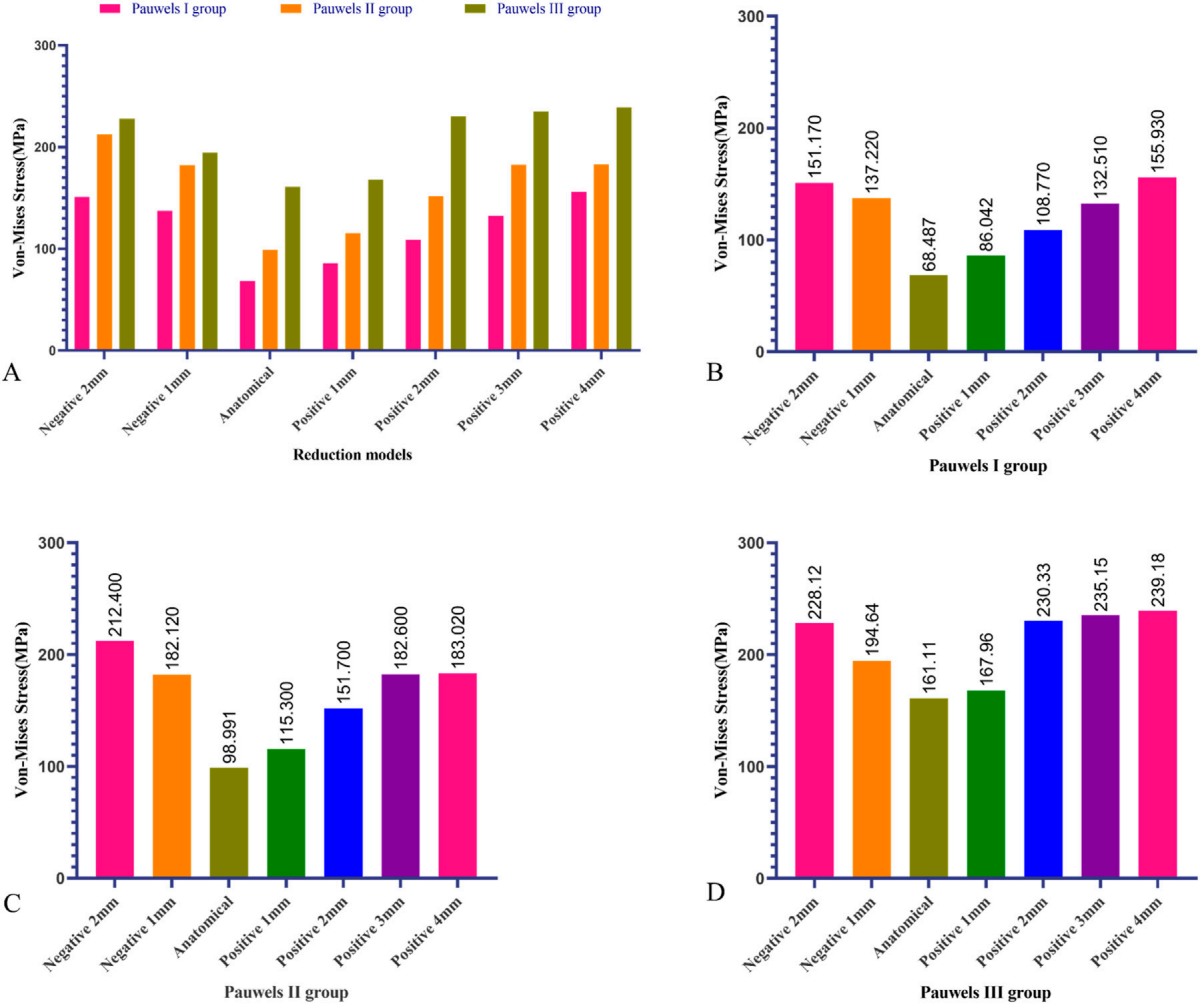
Maximum von-Mises stress of DHS and CS in different Pauwels group with different reduction models. **(A)** Maximum von-Mises stress on the internal fixators varied among the different Pauwels groups, **(B)** Maximum von-Mises stress of DHS and CS of Pauwels I reduction models, **(C)** Maximum von-Mises stress of DHS and CS of Pauwels II reduction models, **(D)** Maximum von-Mises stress of DHS and CS Pauwels III reduction models.

In the Pauwels I, II, and III model groups, the anatomical reduction model displayed the lowest maximum von-Mises stress on the internal fixation, measuring 68.487 MPa, 98.991 MPa, and 161.11 MPa, respectively. In all Pauwels groups, the maximum von-Mises stress concentrated on the internal fixators increased with the escalation of positive reduction. In the Pauwels I group, when positive reduction exceeded 3 mm, the maximum von-Mises stress on the internal fixators surpassed that of the negative reduction model, with the positive reduction 4 mm model exhibiting the highest von-Mises stress at 155.93 MPa. In the Pauwels II group model, when positive reduction exceeded 2 mm, the maximum von-Mises stress on the internal fixators exceeded that of the negative reduction model. In Pauwels III group, when positive reduction exceeded 1 mm, the maximum von-Mises stress on the internal fixators was higher than that of negative reduction model, with the positive reduction 4 mm model exhibiting the highest von-Mises stress at 239.18 MPa (Figure 6).

### 3.2 The maximum von-mises stress on the proximal femur

**FIGURE 7:**
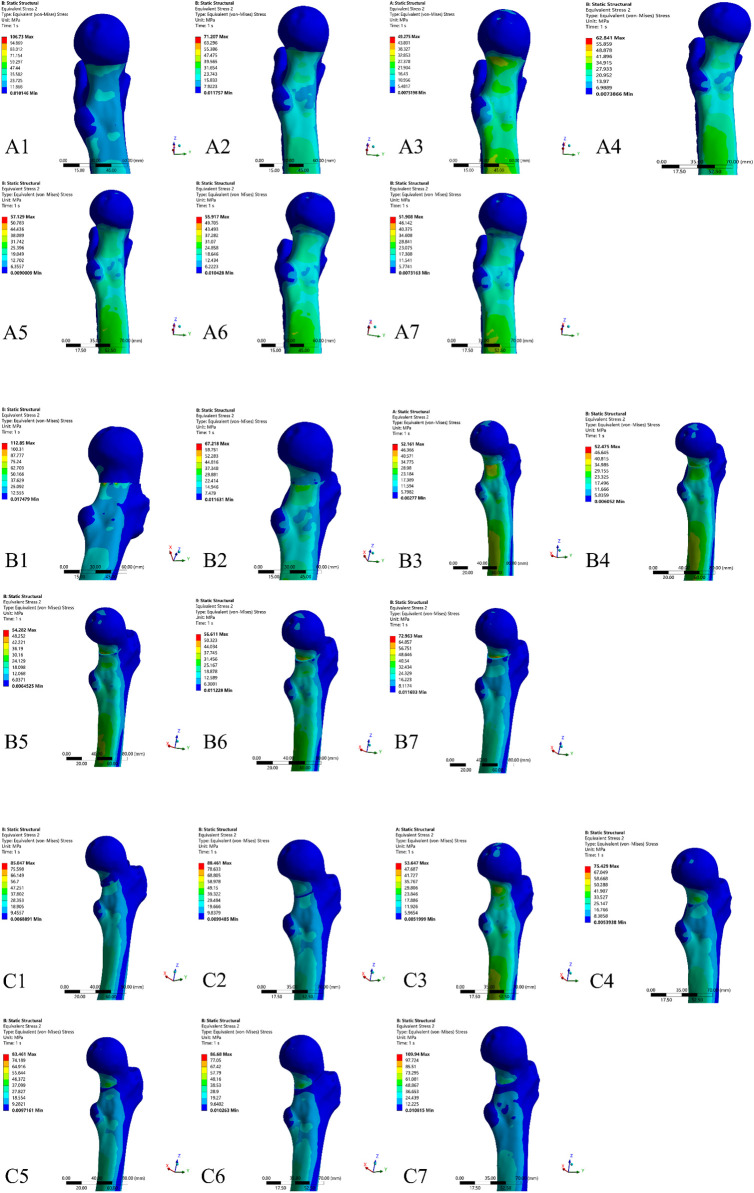
Maximum von-Mises stress nephograms of proximal femur in different Pauwels group with different reduction models. **(A)** Pauwels I group: A1-A2) negative reduction 2 mm and 1 mm model, A3) anatomical reduction model, A4-A7) positive reduction 1 mm, 2 mm, 3 mm and 4 mm model; **(B)** Pauwels II group: B1-B2) negative reduction 2 mm and 1 mm model, B3) anatomical reduction model, B4-B7) positive reduction 1 mm, 2 mm, 3 mm and 4 mm model; **(C)** Pauwels III group: C1-C2) negative reduction 2 mm and 1 mm model, C3) anatomical reduction model, C4-C7) positive reduction 1 mm, 2 mm, 3 mm and 4 mm model.

According to the von-Mises stress nephogram shown in Figure 7, in each Pauwels group, the von-Mises stress distribution on the proximal femur was more evenly distributed at the inferior medial part in the anatomical reduction model compared to both the negative and positive reduction models. Furthermore, in all Pauwels groups, as positive reduction increased, the von-Mises stress gradually concentrated on the medial inferior part of the proximal femur fragment.

**FIGURE 8:**
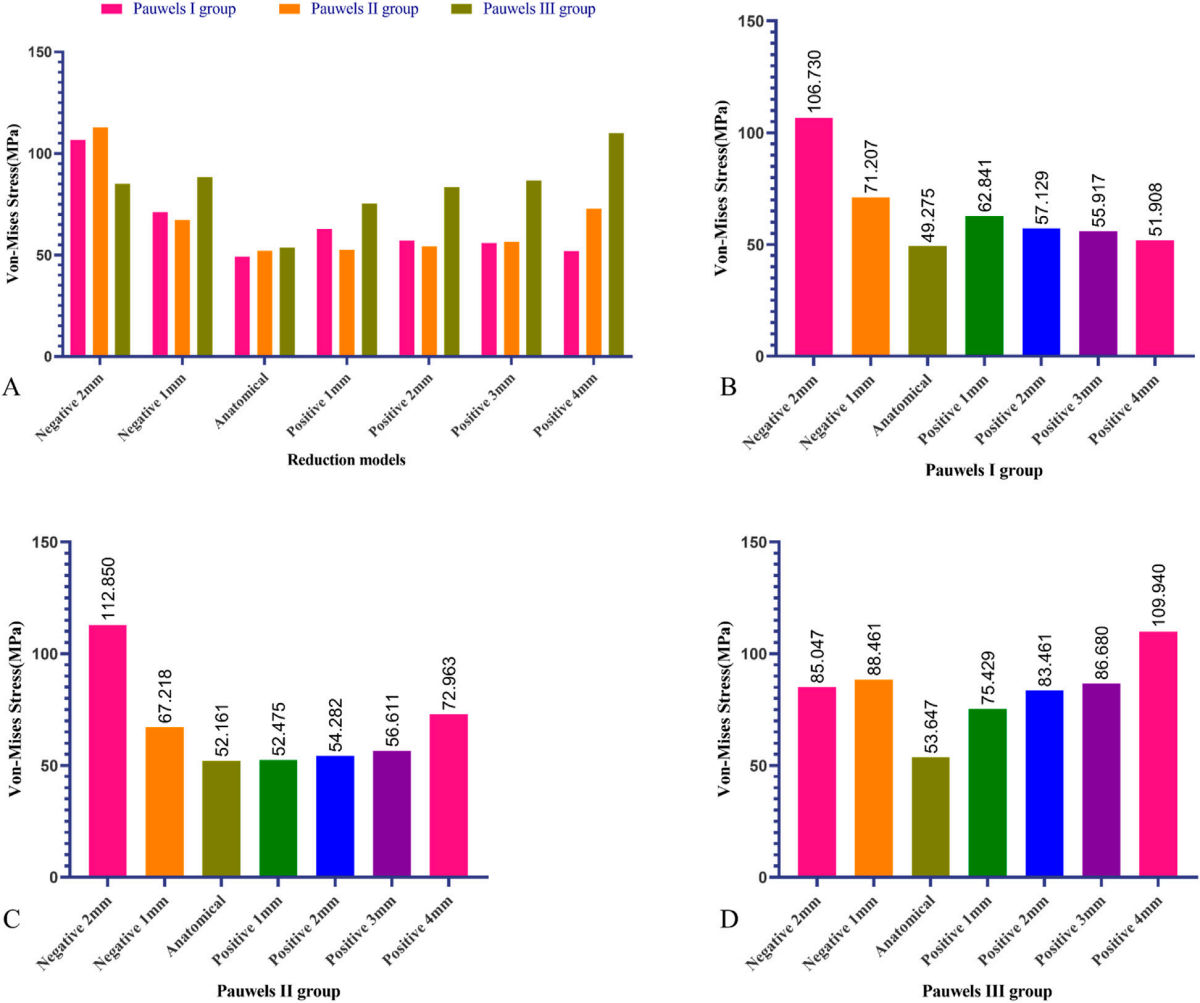
Maximum von-Mises stress of proximal femur in different Pauwels group with different reduction models. **(A)** Maximum von-Mises stress on the proximal femur varied among the different Pauwels groups, **(B)** Maximum von-Mises stress of proximal femur in Pauwels I reduction models, **(C)** Maximum von-Mises stress of proximal femur in Pauwels II reduction models, **(D)** Maximum von-Mises stress of proximal femur in Pauwels III reduction models.

Under a 2100 N load in the Pauwels I, II, and III groups, the anatomical reduction model exhibited the lowest maximum von-Mises stress on the femur, measuring 49.275 MPa, 52.161 MPa, and 53.647 MPa, respectively. In the Pauwels I group, as positive reduction increased, the von-Mises stress of the proximal femur fragment gradually decreased. However, in the Pauwels II and III groups, with increased positive reduction, the von-Mises stress of the proximal femur increased. Furthermore, the highest maximum von-Mises stress on the proximal femur was observed in the negative reduction 2 mm model for the Pauwels I and II groups, measuring 106.73 MPa and 112.85 MPa, respectively. In the Pauwels III group, the highest maximum von-Mises stress was 109.94 MPa, observed in the positive reduction 4 mm model (Figure 8).

### 3.3 The maximum von-mises strain at the fracture site of proximal femur fragment

**FIGURE 9:**
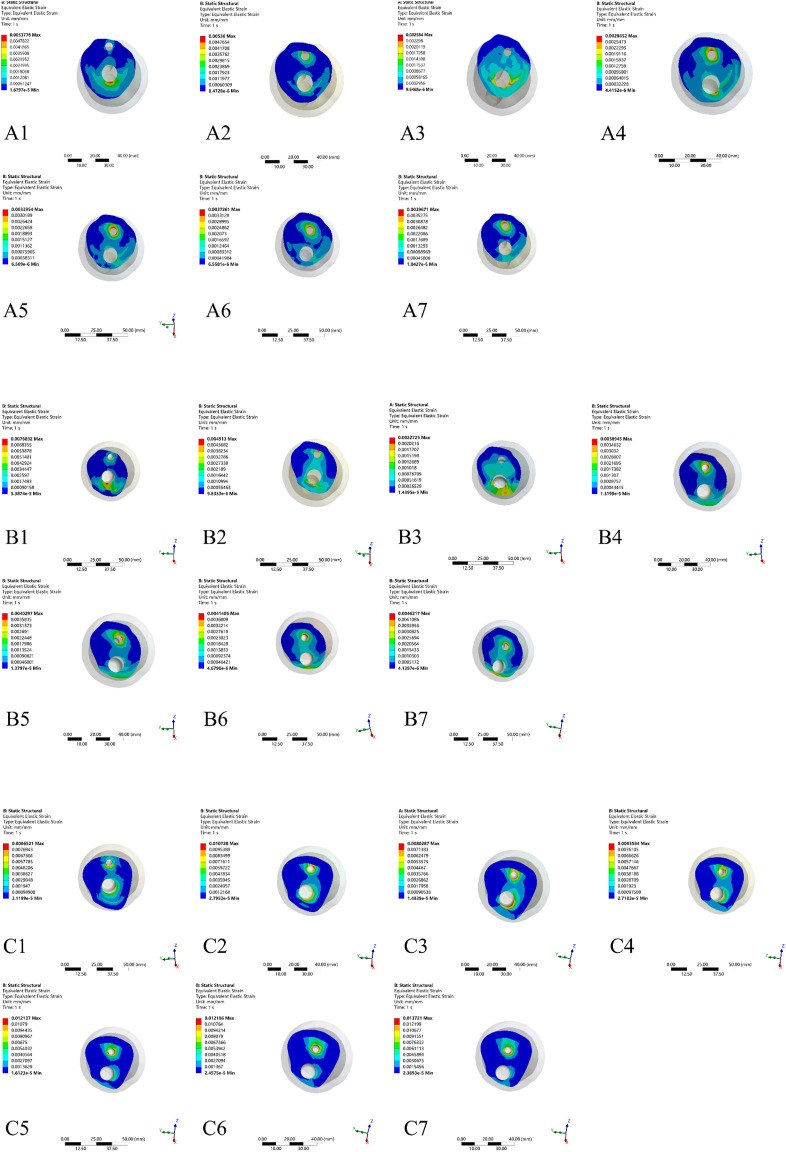
Maximum von-Mises strain nephograms of proximal fragment in different Pauwels group with different reduction models. **(A)** Pauwels I group: A1-A2) negative reduction 2 mm and 1 mm model, A3) anatomical reduction model, A4-A7) positive reduction 1 mm, 2 mm, 3 mm and 4 mm model; **(B)** Pauwels II group: B1-B2) negative reduction 2 mm and 1 mm model, B3) anatomical reduction model, B4-B7) positive reduction 1 mm, 2 mm, 3 mm and 4 mm model; **(C)** Pauwels III group: C1-C2) negative reduction 2 mm and 1 mm model, C3) anatomical reduction model, C4-C7) positive reduction 1 mm, 2 mm, 3 mm and 4 mm model.

As illustrated in Figure 9, the von-Mises strain nephogram at the fracture site of the proximal fragment primarily concentrated on the cancellous region surrounding both the CS and the dynamic compression screw of the DHS. In each Pauwels group, the anatomical reduction model demonstrated a more uniform distribution of von-Mises strain. Additionally, as the positive reduction distance increased, the strain gradually concentrated around the bone adjacent to the CS channel. Especially in the Pauwels III, the strain predominantly concentrated in the cancellous bone around the CS at the fracture site of proximal femur fragment.

**FIGURE 10:**
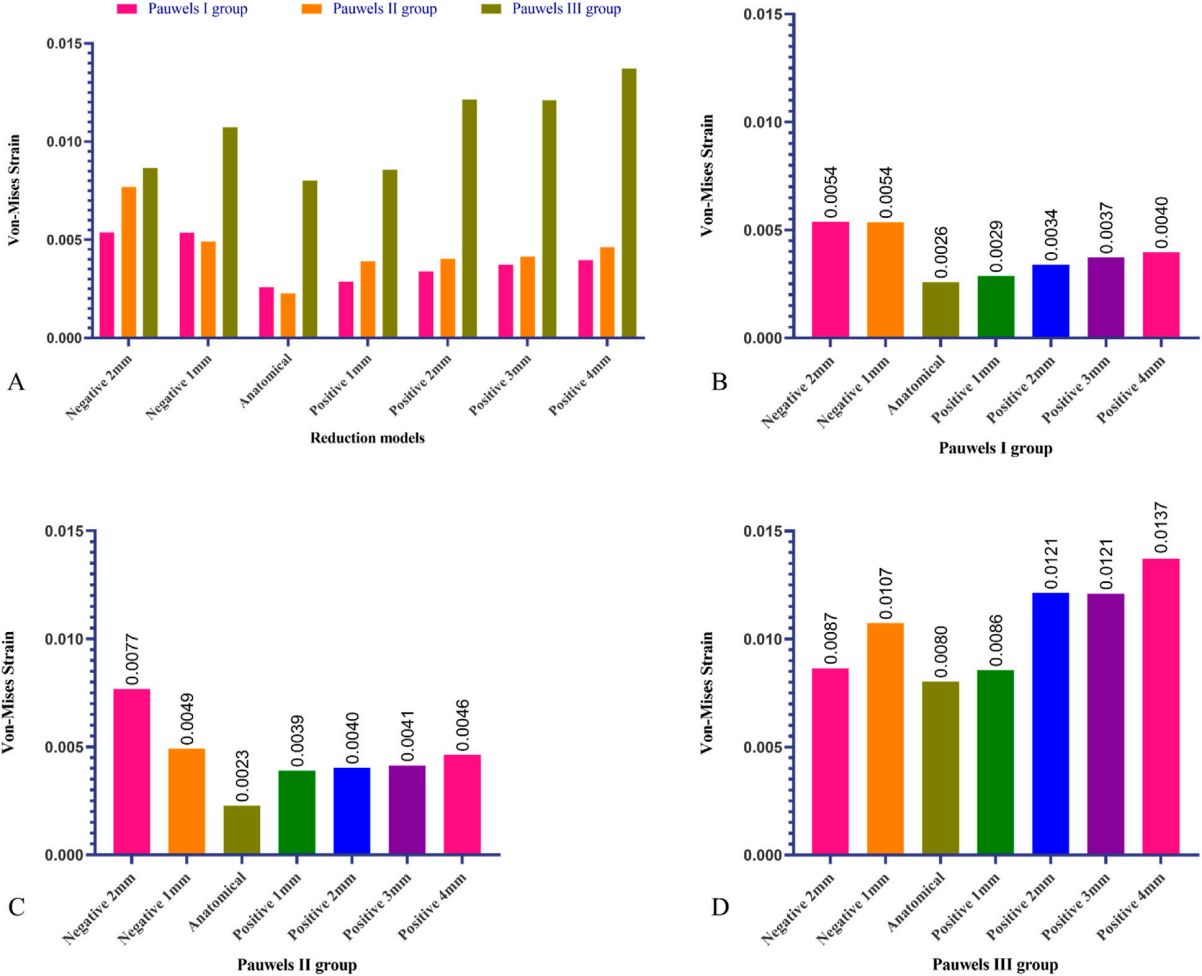
Maximum von-Mises strain of proximal fragment in different Pauwels group with different reduction models. **(A)** Maximum von-Mises strain on the proximal fragment varied among the different Pauwels groups, **(B)** Maximum von-Mises strain of proximal fragment in Pauwels I reduction models, **(C)** Maximum von-Mises strain of proximal fragment in Pauwels II reduction models, **(D)** Maximum von-Mises strain of proximal fragment in Pauwels III reduction models.

The lowest maximum von-Mises strain at the fracture site of proximal femur fragment in the Pauwels I, II, and III group was discovered in the anatomical reduction model with 0.2584%, 0.2273%, and 0.8029% respectively. As the positive reduction increased, the maximum von-Mises strain at the fracture site of proximal femur fragment was increased. Furthermore, the highest maximum von-Mises strain was 0.5378% and 0.7683% in the negative 2 mm reduction model for the Pauwels I and II group. In Pauwels III group, when the positive reduction exceeded 1 mm, the strain of the positive reduction model was higher than that in the negative reduction models. Moreover, the highest maximum von-Mises strain of the Pauwels III group was 1.3721%, which was observed in the positive reduction 4 mm model (Figure 10).

### 3.4 The displacement of the different FNF reduction models after fixation with DHS and CS

**FIGURE 11:**
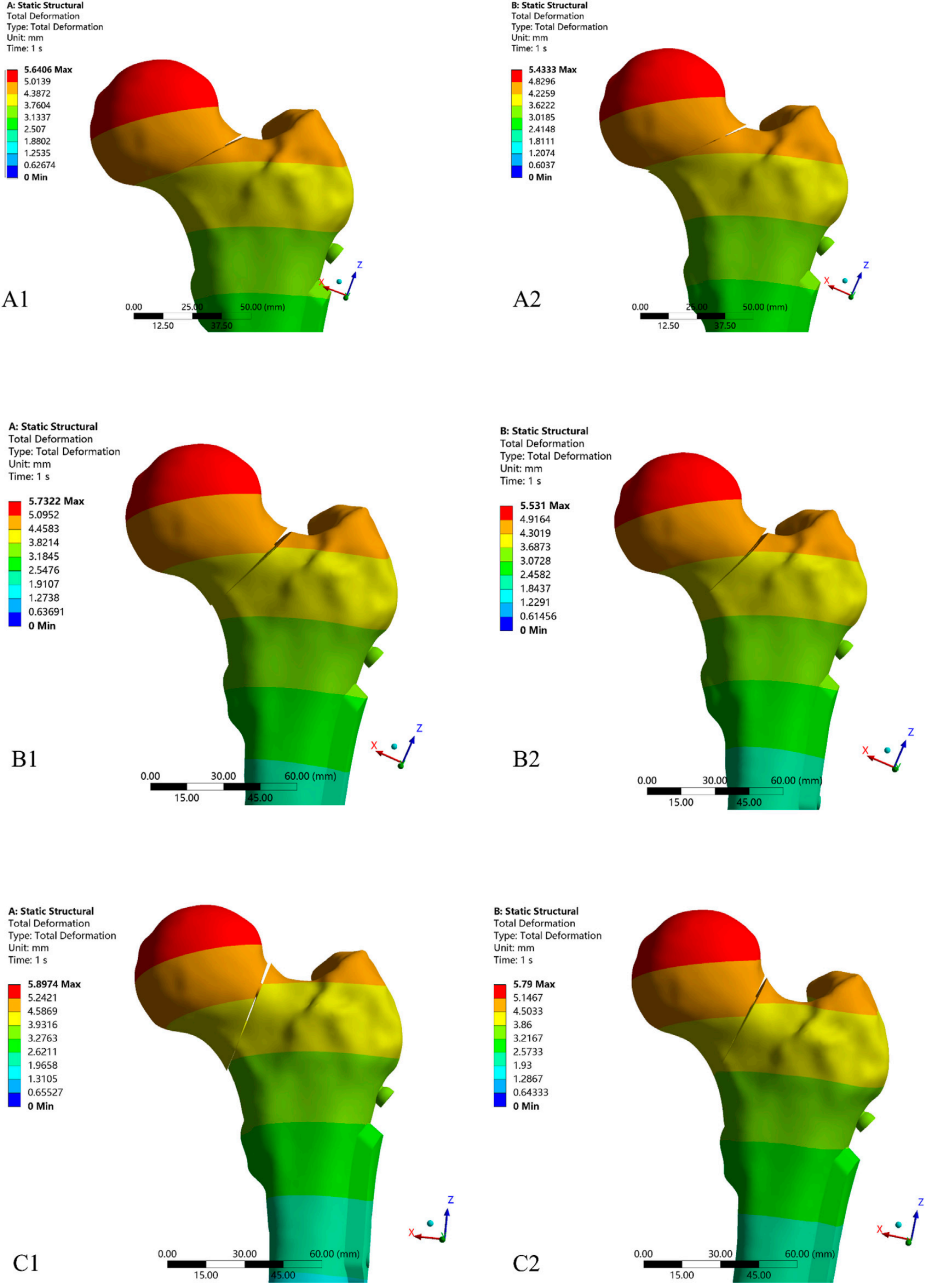
The movement trend of the proximal fragment after proportionally magnified displacement in the anatomical and positive reduction 1 mm models. **(A1)** Anatomical reduction model of Pauwels I group; **(A2)** Positive reduction 1 mm of Pauwels I group; **(B1)** Anatomical reduction model of Pauwels II group; **(B2)** Positive reduction 1 mm of Pauwels II group; **(C1)** Anatomical reduction model of Pauwels III group; **(C2)** Positive reduction 1 mm of Pauwels III group.

As shown in Table 3, negative reduction models exhibited the maximum femoral displacement in each Pauwels group. As the positive reduction increased the femoral displacement was decreased. Furthermore, upon proportionally amplified the displacement results of both the anatomical reduction and the positive 1 mm reduction model within each Pauwels group, it was observed that the anatomical reduction model might transform into the negative reduction model (Figure 11). The results of proximal femoral fragment displacement under the regional coordinate system were presented in Table 4. The negative reduction 2 mm model exhibited the highest displacement in all three axes for Pauwels I and II groups, particularly in the *Y*-axis, where the displacement of the femoral head fragment exceeded that in the other axes. As positive reduction increased, the maximum displacement of the femoral head fragment decreased along each axis in the Pauwels I and II group. However, in the Pauwels III group, the negative reduction 2 mm model showed the highest displacement along the *Y* and *Z*-axes, and the maximum displacement along the *X*-axis was observed in the positive reduction 4 mm model. Furthermore, in all Pauwels groups, as the Pauwels angle increased, the displacement of the femoral head fragment increased in the *Z*-axis.

**TABLE 3:** The femoral displacement after being fixed with DHS and CS in different Pauwels reduction groups (mm).

Groups	Negative		Anatomical	Positive			
	2 mm	1 mm		1 mm	2 mm	3 mm	4 mm
Pauwels I	6.3737	5.7388	5.6406	5.4333	5.2825	5.1225	4.9745
Pauwels II	6.2149	5.7482	5.7322	5.531	5.4374	5.334	5.2532
Pauwels III	6.0132	5.8482	5.8974	5.79	5.7353	5.7074	5.6641

**TABLE 4:** The displacement of the femoral head fragment in different axes based on the regional coordinate system within the different Pauwels group (mm).

Groups		Negative		Anatomical	Positive			
		2 mm	1 mm		1 mm	2 mm	3 mm	4 mm
Pauwels I	*X*-Axis	2.2961	2.2638	2.2807	2.2578	2.2444	2.2205	2.2196
	*Y*-Axis	5.8201	5.1448	5.0345	4.8155	4.6557	4.4903	4.3278
	*Z*-Axis	2.0362	1.8976	1.8689	1.8123	1.7734	1.7259	1.6803
Pauwels II	*X*-Axis	2.3092	2.2659	2.3711	2.2806	2.1765	2.2695	2.1745
	*Y*-Axis	4.9824	4.5199	4.4809	4.2937	4.2563	4.1056	4.0811
	*Z*-Axis	3.5206	3.2802	3.2481	3.1628	3.1098	3.0478	2.9991
Pauwels III	*X*-Axis	2.3534	2.4124	2.4273	2.4274	2.461	2.4721	2.4742
	*Y*-Axis	3.6938	3.5394	3.5799	3.4835	3.4282	3.4049	3.3681
	*Z*-Axis	4.5717	4.4209	4.4607	4.375	4.3215	4.2943	4.2583

### 3.5 Model validation

To validate the reliability and feasibility of the original femoral finite element model developed in our study, we conducted an analysis of the maximum von-Mises stress, axial stiffness, and the von-Mises stress of 8 points on the femoral neck section. We compared these results with previous studies involving FE analysis and cadaver biomechanical research. As shown in Table 5, the validation results of the maximum von-Mises stress of the original intact femur were close to the findings proposed by San Antonio (San Antonio et al., 2012). Additionally, Table 5 presented the results of the axial stiffness of our FE model, which were comparable to the values listed by Papini et al. (2007), falling within the range reported in previous literature (Papini et al., 2007; Miura et al., 2017; Chen et al., 2019; Wang et al., 2021). As depicted in Table 6, concerning the von-Mises stresses at 8 points on the femoral neck cross-section in our FE model, the results at observation points A, B, C, D, G, and H were similar to the data from Zhang et al. (2009) ′FE model 1′ and ′FE model 2′. Furthermore, our results at observation points E and F aligned with the data of ‘FE model 3′and ‘FE model 4′proposed by Zhang in his study (Zhang et al., 2009). The values at our observation points B, C, and G were similar to the outcomes in Matthew et al.'s research (Jian-Qiao Peng et al., 2020).

**TABLE 5:** The maximum von-Mises stress and axial stiffness of the femoral model.

Validation	Compared studies	Outcomes
The maximum von-Mises stress (MPa)	Fu2012	22
	San (1) 2012	17.95
	San (2) 2012	17.49
	San (3) 2012	18.05
	Own	17.649
Axial stiffness (KN/mm)	Papini2007	0.757 ± 0.264
	Jian chen 2019	0.54
	Miura2017	1.566(FEA)/1.28(Mechanical test)
	Wang Kaiyang2021	1.32123(FEA)/1.12911(Measurement)
	Own	0.8344

**TABLE 6:** The maximum von-Mises stresses at 8 points on the femoral neck cross-section of the FE model.

Compared studies		The maximum von-mises stresses at 8 points on the femoral neck cross-section (MPa)							
		A	B	C	D	E	F	G	H
Matthew 2020	FE	1.7486	1.0082	1.5609	0.4760	2.9542	2.0889	1.1033	0.6937
	Cadaver	2.0805	0.8395	2.4638	0.2555	3.2609	2.7923	1.3688	0.2190
Zhang G 2009	FE model 1	5.6195	2.7540	2.7657	2.7075	2.7044	2.6721	2.6699	5.6195
	FE model 2	4.6121	2.5308	2.5240	2.6403	2.6073	2.6197	2.6159	4.6121
	FE model 3	13.4386	6.6241	6.5349	6.9777	6.9740	6.8769	6.8681	13.4386
	FE model 4	22.9186	8.1142	8.3652	8.3234	8.3628	8.3917	8.3884	22.9186
Own		6.6864	2.5484	2.5166	3.5549	7.3338	6.0198	2.2854	4.2151

## 4 Discussion

In clinical practice, achieving high-quality reduction and stable internal fixation is widely recognized as crucial in treating FNF (Ai et al., 2013). Anatomical reduction is the goal pursued by surgeons, but it can be challenging in cases where the fracture line is more vertical, or the fracture is highly comminuted. However, repeated attempts at reduction to achieve anatomical alignment may compromise the blood supply to the femoral neck fracture site, thereby elevating the risk of postoperative complications (Su et al., 2011; Xiong et al., 2019; Zhuang et al., 2019). The concept of positive reduction has gained acceptance among orthopedic surgeons as it can achieve comparable clinical results to anatomical reduction in FNF. However, there is still a lack of research on the biomechanical behavior of different reduction configurations based on the Gotfried positive reduction concept. Which promoted us to conduct a FE analysis to quantitatively explored the biomechanical performance of positive reduction in FNF.

According to our results, the anatomical reduction model exhibited the lowest maximum von-Mises stress in both internal fixators and the proximal femur across all Pauwels groups, suggesting a potentially more stable environment for FNF healing and a lower risk of complications (Johansson et al., 2001). Especially for patients under 65 years who are engaged in frequent daily activities, anatomical reduction implies a better prognosis after surgery (Li and Cole, 2015). However, our study found that the positive reduction could achieve a similar biomechanical stability as the anatomical reduction model, but there were some specific limitations. In the Pauwels I group, as the positive reduction increased, the maximum von-Mises stress in the proximal femur decreased. This observation may be attributed to the smallest fracture angle in the Pauwels I group, where the positive reduction facilitating the embedding of the proximal fragment into the distal fracture site, sharing part of the von-Mises stress borne by the internal fixators. With the increased in positive reduction in the Pauwels I group, the advantage of von-Mises stress sharing by positive reduction gradually decreased, and the von-Mises stress borne by the internal fixation was increased. When positive reduction exceeded 3 mm, the maximum von-Mises stress on the internal fixators exceeded that in the negative reduction model. In the Pauwels II group, as positive reduction increased, the maximum von-Mises stress both on the proximal femur and internal fixators were gradually increased. When positive reduction exceeded 2 mm, the maximum von-Mises stress on the internal fixators increased to 182.6 MPa, surpassing the stress observed in the negative reduction 1 mm model. In Pauwels III, when positive reduction exceeded 1 mm, both the maximum von-Mises stress on the proximal femur and internal fixators increased, surpassing the levels observed in the negative reduction 1 mm model and the positive 4 mm model displayed the highest maximum von-Mises stress on the internal fixators. The primary objective of positive reduction is to redistribute vertical stress by improving cortical support on the medial side fragment of FNFs. However, excessive stress concentration at the internal fixation or fracture site is highly likely to result in fatigue failure of the fixation device or failure in fracture reduction. Based on our result of von-Mises stress, we found that instead of enhancing the stability of internal fixation, excessive positive reduction would lead to higher stress on the internal fixators in all the Pauwels group. Additionally, the higher complication incidence in FNFs was directly associated with the inadequate fracture reduction (Araujo et al., 2014). With the increase in the Pauwels angle, the fragments at the FNF site may become more comminuted (Han et al., 2022), influencing the supportive effect of positive reduction on the medial cortex of the femoral neck, and leading to the internal fixation bearing greater stress.

According to previous literature, the risk of bone micro-damage would be increased when the strain magnitude in response to mechanical loading exceeds 4,000 με (1 με = 0.0001%) (Frost, 1987). In our study, we adopt a threshold of 0.4% for our strain analysis. Regarding the von-Mises strain on the fracture site of proximal femur fragment, the anatomical reduction group displayed the lowest maximum von-Mises strain among the three Pauwels groups. As the positive reduction increased, the strain shifted to concentrate around the cancellous bone adjacent to the CS hollow. In Pauwels I group, when positive exceeded 3 mm the maximum von-Mises strain was 0.397%, approaching the bone micro-damage threshold. In Pauwels II group, when positive exceeded 2 mm the maximum von-Mises strain was 0.403%, surpassing the bone micro-damage threshold. Furthermore, the highest maximum von-Mises strain was 0.5378% and 0.7683% in the negative 2 mm reduction model for the Pauwels I and II group. However, in Pauwels III group, the maximum von-Mises strain in all reduction models surpassing the bone micro-damage threshold. Especially, when the positive reduction exceeded 1 mm, the strain of the positive reduction model was higher than that in the negative reduction models. The higher strain on the bone implies a greater risk of bone micro-damage and fracture (Forwood and Turner, 1995). In our study, as positive reduction and the Pauwels angle increased, the strain concentrated around the cancellous bone surrounding the screw also increased. This indicates a higher risk of bone micro-damage and deformation in this region, raising the risk of internal fixation failure as the screw cutting out from the proximal femoral fragment and elevating the possibility of reoperation for elderly or osteoporotic FNF patients after screw internal fixation (Dolatowski et al., 2019; Cui et al., 2020).

In our FE study, the highest femoral displacement was observed in the negative reduction FNF models in each Pauwels group. This suggests that negative reduction models might be insufficient to provide stability (Wang G. et al., 2019). In each Pauwels group, the positive reduction model exhibited lower femoral displacement than both the negative and anatomical reduction groups. Additionally, as the distance of positive reduction increased, the post-fixated femoral displacement correspondingly decreased. This finding differs from some previous FE studies that argued the displacement of the femur should increase with growing positive reduction (Wang G. et al., 2019; Fan et al., 2023). However, our finding is consistent with the theory of positive reduction, which reduce the femoral head fragment displacement by achieving cortical buttress support from the medial inferior fragment (Zhang and Chang, 2013). Furthermore, proportional amplification of the displacement results for both the anatomical reduction and positive 1 mm reduction models reveals that the anatomical reduction model might transform into a negative reduction model in our finite element simulations and this aligns with a previous study (Zhu et al., 2022). This suggests that the cortical support from the distal fragment in the positive reduction model functions similarly to a medial buttress plate (Zhao et al., 2021a). Fracture healing requires a stable mechanical environment, both shear force and varus stress at the fracture end can impact the healing processes (Bartonícek, 2001). As previous research, common complications of FNFs include femoral head avascular necrosis, fracture non-union, femoral neck shortening, and varus deformity, constituting 6.6%, 19.3%, 66%, and 39%, respectively (Parker et al., 2007; Zlowodzki et al., 2008; Loizou and Parker, 2009). Among these complications, hip varus displacement is a significant indicator of a poor prognosis for FNFs (Nowotarski et al., 2012). Based on the results of proximal femoral fragment displacement using the regional 3D coordinate system, it was observed that as positive reduction increased, the maximum displacement of the femoral head fragment decreased along each axis in the Pauwels I and II groups. However, in the Pauwels III group, as positive reduction increased, the displacement in the *X*-axis increased. Furthermore, with the Pauwels angle increased, the displacement of the femoral head fragment increased in the *Z*-axis, suggesting a greater potential for hip varus in Pauwels III group. These results suggest that the stability of positive reduction is influenced by the Pauwels angle. The advantage of positive reduction gradually diminishes as the angle increases, and excessive positive reduction might exacerbate varus displacement and additional rotation deformity in Pauwels III.

Although our FE analysis relied on a single dataset, the validation results of the femur model indicated biomechanical properties that were generally consistent with previous literature. This suggests that our femoral FE model could offer reliable and valuable results for this study. Based on our results, in the Pauwels I group, it is advisable to limit positive reduction to 3 mm or below, as this could achieve an effect similar to the anatomical reduction model. Similarly, in the Pauwels II group, positive reduction should be restricted to not exceed 2 mm. For the Pauwels III group, positive reduction should be limited to 1 mm or below, demonstrating a biomechanical behaviour closer to the anatomical reduction model. Furthermore, negative reduction should be avoided as much as possible in all Pauwels groups.

There are still some limitations in our research. Firstly, our FE model in this study was based on a single intact human femoral CT dataset, which is similar to the other FE analyses (Wang G. et al., 2019; Fan et al., 2023). The effect of positive reduction may vary among patients due to multifactorial differences such as height, weight, age, and gender. To further confirm our result, a multicenter retrospective clinical study and comprehensive cadaveric biomechanical research on positive reduction should be conducted. Secondly, like other FE analyses, in our study the material of models was assumed to be homogeneous, continuous, and isotropic (Ding et al., 2021; Zhu et al., 2022; Fan et al., 2023). However, human bone is actually an anisotropic heterogeneous material. Future model construction could be enhanced by incorporating more realistic bone properties. Besides this, the fragments of FNF should be more comminuted in the real clinical situation, which is impossible to simulate in our intact FE model. Nonetheless, as a preliminary quantified investigation of positive reduction, these assumptions are deemed reasonable. It is necessary to rebuild more accurate bone fragments to better mimic the real situation of fracture sites in future studies.

## 5 Conclusion

Excessive positive reduction may increase the risk of FNF failure after internal fixation. From a biomechanical stability perspective, positive reduction should be limited within 3 mm or below in the Pauwels I group, should be restricted to not exceed 2 mm in the Pauwels II group and should not exceed 1 mm in the Pauwels III group. Negative reduction should be avoided in all types of Pauwels angles.

## Data Availability

The raw data supporting the conclusions of this article will be made available by the authors, without undue reservation.

## Abbreviations

FE, finite element; FNF, femoral neck fracture; AP, anteroposterior; DHS, dynamic hip screw; CS, cannulated screws; CT, computed tomography; AO/ASIF, association for the study of internal fixation.
